# Modification of PSf/SPSf Blended Porous Support for Improving the Reverse Osmosis Performance of Aromatic Polyamide Thin Film Composite Membranes

**DOI:** 10.3390/polym10060686

**Published:** 2018-06-20

**Authors:** Li-Fen Liu, Xing-Ling Gu, Xin Xie, Rui-Han Li, Chun-Yang Yu, Xiao-Xiao Song, Cong-Jie Gao

**Affiliations:** 1 Center for Membrane and Water Science and Technology, Ocean College, Zhejiang University of Technology, Hangzhou 310014, China; 13588348159@163.com (X.-L.G.); 15958041452@163.com (X.X.); 15857112095@163.com (R.-H.L.); gaocj@zjut.edu.cn (C.-J.G.); 2 Collaborative Innovation Center of Membrane Separation and Water Treatment of Zhejiang Province, Hangzhou 310014, China; 3 State Key Laboratory of Metal Matrix Composites, School of Chemistry & Chemical Engineering, Shanghai Jiao Tong University, 800 Dongchuan Road, Shanghai 200240, China; chunyangyu@sjtu.edu.cn

**Keywords:** sulfonated polysulfone, blending porous support, doping content of SPSf, polyamide TFC membrane, pore nature, reverse osmosis performance

## Abstract

In this study, modification of polysulfone (PSf)/sulfonated polysulfone (SPSf) blended porous ultrafiltration (UF) support membranes was proposed to improve the reverse osmosis (RO) performance of aromatic polyamide thin film composite (TFC) membranes. The synergistic effects of solvent, polymer concentration, and SPSf doping content in the casting solution were investigated systematically on the properties of both porous supports and RO membranes. SEM and AFM were combined to characterize the physical properties of the membranes, including surface pore natures (porosity, mean pore radius), surface morphology, and section structure. A contact angle meter was used to analyze the membrane surface hydrophilicity. Permeate experiments were carried out to evaluate the separation performances of the membranes. The results showed that the PSf/SPSf blended porous support modified with 6 wt % SPSf in the presence of DMF and 14 wt % PSf had higher porosity, bigger pore diameter, and a rougher and more hydrophilic surface, which was more beneficial for fabrication of a polyamide TFC membrane with favorable reverse osmosis performance. This modified PSf/SPSf support endowed the RO membrane with a more hydrophilic surface, higher water flux (about 1.2 times), as well as a slight increase in salt rejection than the nascent PSf support. In a word, this work provides a new facile method to improve the separation performance of polyamide TFC RO membranes via the modification of conventional PSf porous support with SPSf.

## 1. Introduction

Reverse osmosis (RO), with excellent separation performance, is one of the most efficient membrane filtration techniques and has been widely used to produce fresh water from saline water [1,2,3,4,5,6]. The reverse osmosis membrane is the core of the RO technology. At present, aromatic polyamide thin film composite (TFC) membrane fabricated by interfacial polymerization (IP) has been the mainstream product in the RO membrane market.

The conventional polyamide TFC RO membrane consists of three layers. The bottom layer is a non-woven fabric support, the middle layer is generally polysulfone (PSF) or polyethersulfone (PES) porous support, and the top skin layer is the aromatic polyamide film. This composite structure makes it convenient to tailor the porous support layer and polyamide film independently to optimize the RO membrane’s properties. Factors including chemical and physical properties of the porous support, the component of the monomer used in IP process, catalysts and additives, the craftsmanship of IP and post-treatment were adjusted to reconcile the natures of the polyamide TFC membrane, such as chemical structure, physical properties and separation performance [7,8].

It has been widely accepted that the polyamide thin film determines the permeability and the selectivity of the final composite RO membrane and most research on the polyamide skin layer were devoted to improving permeability, selectivity, and fouling-resistance of the membrane by surface modification and/or nanoparticles incorporation [9,10,11,12,13,14,15,16,17]. Actually, the physical morphology and chemical properties of the porous support deeply influenced the formation of the polyamide thin film [18,19,20]. Yakavalangi et al. [21] revealed that the porosity and pore size of the ultrafiltration (UF) substrate could be adjusted by changing the polymer concentration and solvent in the casting solution, which further influenced M-phenylenediamine (MPD) distribution on the support and its diffusion rate into the trimesoyl chloride (TMC) solution. Ghosh and Hoek [22,23] reported the relationship between MPD diffusivity and solubility in the organic phase during the interfacial polymerization of MPD and TMC, and further demonstrated that the relatively porous and hydrophobic support contributed to fabricate rougher, thicker, and more permeable RO membranes due to the formation of less polyamide within the pores [23]. However, some researchers thought that the larger pore size of the porous support made less permeable and more selective polyamide [24,25]. Furthermore, the performance of polyamide thin film was related to the hydrophilicity of the substrate and the dip time of MPD solution on the substrate [26]. It was thus proposed that the more hydrophilic support was beneficial for the formation of polyamide films with higher permeability and salt rejection at the same MPD solution dip time.

So far, different polymers have been applied to produce the porous supports by the conventional phase inversion (PI) method, such as polyethersulfone (PES), polycarbonate (PC), polysulfone (PSf), polypropylene, poly(styrene-co-acrylonitrile), poly(phthalazinone ether sulfone ketone) (PPESK), polyacrylonitrile (PAN), and so on [27,28,29,30]. Polysulfone has some advantages, including wide availability, relatively low cost, and fairly stability, thus it is presently the most popular polymer for preparation of porous supports in the fabrication of polyamide TFC RO membranes. However, polysulfone is relatively hydrophobic compared to most of the other polymers, which directly influences the distribution of MPD on the support and further impacts the interfacial polymerization with TMC [31,32,33,34,35,36]. It is worth noting that sulfonated polysulfone (SPSf) presents higher hydrophilicity than polysulfone and maintains other merits of polysulfone. Obviously, SPSf has good potential to improve the hydrophilicity of the PSf porous support by blending with PSf to fabricate PSf/SPSf blended membranes via the conventional phase inversion method.

In this study, consequently, SPSf was used to modify the conventional PSf ultrafiltration and the composition of the casting solution, including the solvent type, polymer concentration, and doping content of SPSf, was adjusted to regulate the pore nature and the surface properties of the PSf/SPSf blended membrane. In order to further investigate the effect of the porous support on the performance of TFC RO membranes, the polyamide composite RO membranes were prepared synchronously on the resultant porous supports via interfacial polymerization. Scanning electron microscopy (SEM) and Image J were applied to characterize the surface morphology and pore structure of the supports. Besides that, the hydrophilicity, roughness, and pure water flux of the PSf/SPSf support were measured. Their effects on the formation of the polyamide skin layer and the performance of the final polyamide RO membrane were further evaluated.

## 2. Materials and Methods

### 2.1. Materials and Reagents

**Scheme 1:**
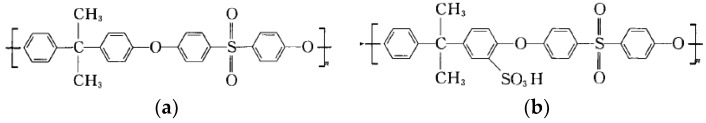
Chemical structure of polysulfone and sulfonated polysulfone, (**a**) PSf; (**b**) SPSf.

Polysulfone (PSf, UDEL P-3500,) and sulfonated polysulfone (SPSf, DS: 10%) descript as Scheme 1, purchased from Solvay (Alpharetta, GA, USA), were first dried at 80 °C for 12 h prior to use. *N*,*N*-dimethylformamide (DMF, >99%), *N*,*N*-dimethylacetamide (DMAc, >99%), *N*-methyl-2-pyrrolidone (NMP, >99%) were purchased from Aladdin Reagent Co. Ltd. (Shanghai, China) and were used as solvents to fabricate the UF blended membrane as the porous support for the composite RO membrane. M-phenylenediamine (MPD, >99.5%), trimesoyl chloride (TMC, >99.5%) and aqueous phase additives including triethyl amine (TEA), (+)-10-champhor sulfonic acid (CSA), and sodium dodecyl sulfate (SDS) were purchased from J&K Scientific Ltd. Beijing, China, and were used to prepared polyamide layer on the porous support via interfacial polymerization. All reagents were of analytical grade and used directly without further purification. Deionized (DI) water was self-produced in-lab through a two-stage RO system.

### 2.2. Fabrication of Porous Support Membrane

The porous support membranes, including the conventional PSf UF membrane (PSf concentration in the range of 16 wt % to 24 wt %) and the PSf/SPSf blended membrane, were fabricated by non-solvent-induced phase separation. For the PSf/SPSf blended support, certain amount of dry polysulfone and sulfonated polysulfone according to Table 1 were added into a sealed round-bottom flask and then a certain amount of solvent (DMAc, NMP, or DMF) was added to dissolve the blending polymers. The solution was stirred for several hours (about 4 h) to insure complete dissolution and was then degassed overnight to remove bubbles. The degassed polymer dope was cast on a polyester nonwoven fabric attached to a glass plate by a knife blade of 0.2 mm gap, then the whole assembly was immersed immediately into a room temperature coagulation bath. The precipitated PSf/SPSf blended support was taken out of the coagulation bath, rinsed with de-ionized water to remove residual impurities, and was finally stored in wet condition.

### 2.3. Fabrication of Polyamide TFC Reverse Osmosis Membrane

Polyamide TFC RO membranes were prepared by interfacial polymerization of MPD and TMC solutions as provided in Table 2. First, MPD aqueous solution was poured into the support membrane formed previously until total soaking. The excess MPD solution was removed from the support surface with a rubber roller 2 min later and then air-dried at ambient temperature until no residual liquids remained. Subsequently, the TMC–hexane solution was poured into the MPD-saturated support, then maintained for 40 s, followed by washing with n-hexane. Finally, the resultant nascent polyamide TFC membrane was heat cured at 60 °C for 5 min for further polymerization. The obtained polyamide TFC RO membranes were washed in DI water (conductivity ≤5 μS/cm) and stored in NaHSO_3_ solution (1.0 wt %) for later use.

### 2.4. Permeability Tests

**Figure 1:**
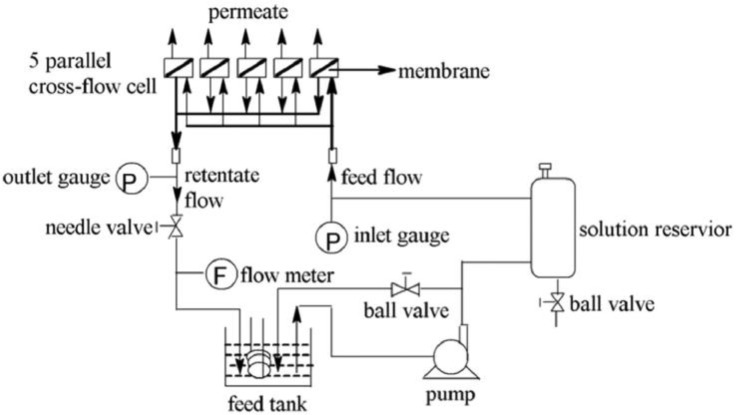
Schematic drawing of the setup for permeation testing of the RO membrane.

Pure water permeability of the UF support membrane was determined by a stainless steel dead-end filtration system (Figure 1). Membrane coupons with an effective area of 12 cm^2^ were equilibrated with DI water at room temperature and under pressure of 0.2 MPa for 0.5 h and then under pressure of 0.1 MPa. Each membrane was tested at least three times and the average value defined the water fluxes of the supports which were calculated with the equation as below:

*Jw* = *V*/(*Am* × *t*) (1)

*Jw*, *V*, *Am*, and *t* represent pure water flux (L/m^2^·h), volume of the permeate (L), membrane area (m^2^), and time of permeation test (h), respectively.

The membrane permeability coefficients *A* (μm/MPa·s) were defined by normalizing the pure water flow by the applied pressure and membrane area as follows:

*A* = *Jw*/(Δ*P* − Δπ) (2)

Δ*P* is the applied pressure and Δπ is the osmotic pressure in the test cell.

For the polyamide TFC RO membrane, the water permeability and salt rejection performances were tested at least three times using a homemade stainless steel cross-flow membrane filtration unit. The membrane samples were picked to eliminate samples with deficits before testing. Membrane coupons were soaked in DI water for at least 0.5 h to remove the impurities before loading to the test unit. The membrane was equilibrated at room temperature under pressure of 1.55 MPa for 1 h. After flux had been maintained steadily for 30 min, the selectivity of the resultant polyamide TFC RO membranes was characterized by measuring rejection of 2000 ppm NaCl solution at the pressure of 1.55 MPa for 1 h.

The salt permeance (*B*) was determined by the following equation:

*R* = [1 − (*C_P_*/*C_F_*)] × 100 (3)

*B* = *Jw*(1 − *R*)/*R* (4)

*R*, *C_P_*, and *C_F_* represent the salt rejection and salt concentrations in the permeate and feed, respectively.

### 2.5. Characterization of PSf/SPSf Porous Support and Polyamide RO Membrane

The viscosity of the casting solution for the fabrication of the porous support membrane was tested by a Rotor Viscosimeter (Brookfield, DV-2T, Middleboro, MA, USA).

The thickness and surface morphology of the porous support membrane and corresponding polyamide TFC RO membrane was characterized by scanning electron microscopy (SEM, S-4700 Hitachi, Tokyo, Japan). The membrane samples were broken in liquid nitrogen to obtain a slick surface. Then, the fractured parts were placed on a holder and coated by gold after drying in a desiccator. The SEM images were taken at 15 KV. The observed SEM images of porous support membrane were further processed with Image J software to calculate surface porosity and the membrane surface pore diameter was calculated according to Equation (5) [21]:

(5) $$Rm=\sqrt{\frac{\left(2.9-1.75\varepsilon \right)\times 8\eta lQ}{\varepsilon \times A\times \Delta P}}$$

where *A* and ε represent the effective area (m^2^) and the porosity (%) of the support, respectively. η is the viscosity of water, *Q* is the volume of the permeate (m^3^/s), and Δ*P* is the test pressure (0.1 MPa).

Atomic force microscope (AFM, Bruker Dimension Icon, Billerica, MA, USA) equipped with an antimony-doped silicon cantilever was used to analyze the surface roughness of both the porous support membrane and the polyamide RO membrane. The cantilever length is 125 μm with a force constant of 42 Nm^−1^. Air-dried membrane samples were fixed on a specimen holder and 5 μm^2^ areas were scanned with the rate of 0.799 Hz by tapping mode in air. Roughness was quantified by the root mean square (RMS) roughness.

The surface hydrophilicity of both the porous support and the polyamide RO membrane were measured by contact angle goniometer (OCA50AF, Dataphysics, Hamburg, Germany) with equilibrium sessile drop method of de-ionized water on dried membrane surfaces. At least twelve equilibrium contact angles were obtained for each sample and the average value was determined without the minimum and maximum.

## 3. Results

The conventional PSf ultrafiltration membrane was successfully modified by doping with SPSf via the phase inversion method and the polyamide RO membranes were further prepared based on the resultant PSf/SPSf blended porous support via interfacial polymerization technology. The effects of the SPSf/PSf porous support on the structure and performance of polyamide RO membrane were investigated systematically by adjusting the composition of the casting solution including solvent, polymer concentration, and doping content of SPSf. Several results were obtained, as follows.

### 3.1. Effects of Solvent and Polymer Concentration on the Porous Support and RO Membranes

NMP, DMAc, and DMF were selected as the solvents of the polymer PSf with concentrations in the range of 16–24 wt % to prepare the casting solution, on the basis of which, the effects on the porous supports and corresponding RO membranes were investigated in detail.

#### 3.1.1. Morphology of the Membranes

**Figure 2:**
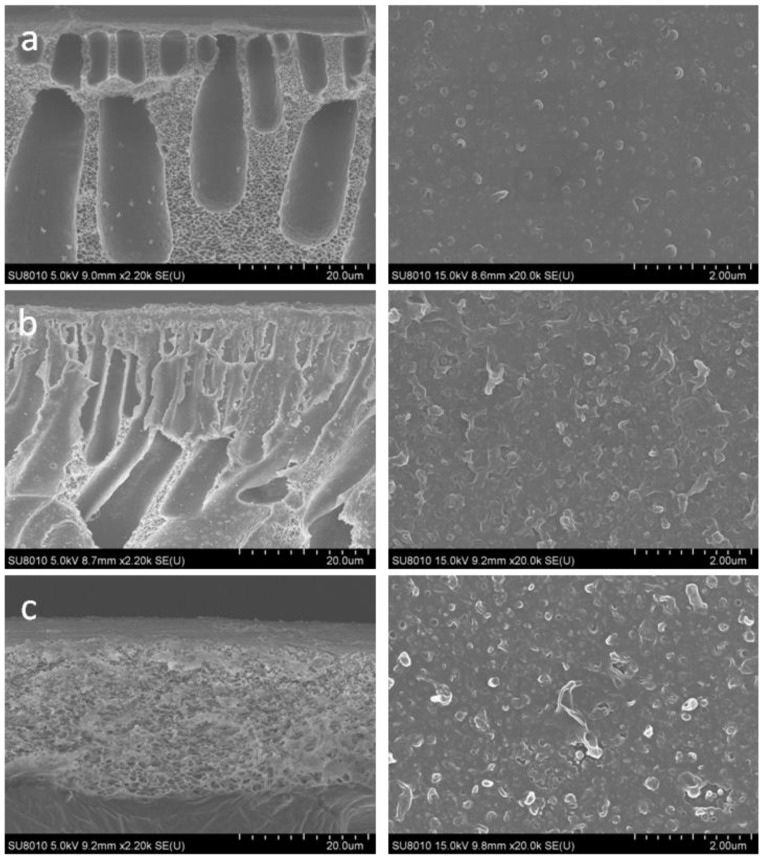
SEM images of the overall cross-section (left) and the membrane surface (right) of TFC RO membrane with 20 wt % PSf in different solvents; (**a**) NMP, (**b**) DMAc, and (**c**) DMF, respectively.

SEM was used to observe the surface cross-section morphology of the prepared PSf porous supports and corresponding TFC RO membranes, as shown in Figure 2. The results showed that the cross-sections of the supports prepared from NMP and DMAc exhibited finger-like structures, while that of DMF exhibited sponge-like structures. All of the RO membranes based on the three kinds of supports presented typical ridge-and-valley structures, but the later RO membrane had a relatively rougher surface than the two former membranes.

#### 3.1.2. Membrane Separation Performances

**Figure 3:**
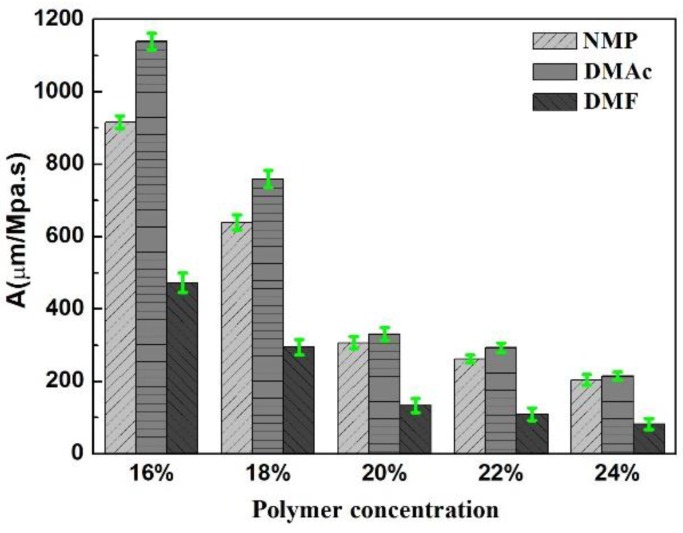
Permeability coefficients of the porous supports in different solvents.

**Figure 4:**
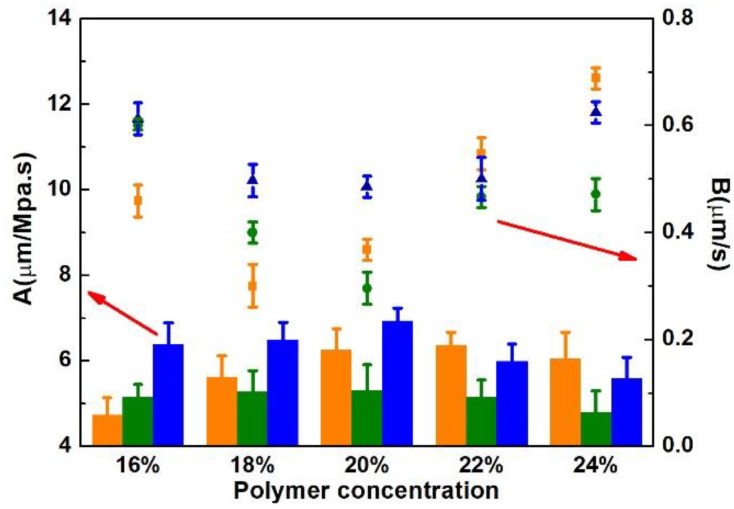
Effects of different solvents and polymer concentrations on the performance of the RO membranes. Column and solid scatters denote A and B, respectively. Orange, green, and blue present NMP, DMAc, and DMF, respectively.

The separation performances of the porous supports and polyamide RO membranes were evaluated and the results showed that DMF and 16 wt % of PSf endowed the porous support membrane with the highest flux (Figure 3), but only the support prepared with DMF and 20 wt % PSf were more suitable for fabrication of the polyamide TFC RO membrane (Figure 4).

### 3.2. Effects of Doping Content of SPSf on the Porous Support and RO Membrane

The doping content of SPSf in the casting solution was adjusted to regulate the structure and performance of the PSf/SPSf blended support with the purpose of improving the performance of the polyamide TFC RO membrane.

#### 3.2.1. Viscosity of the Casting Solution, Thickness and Pore Nature of the Porous Support

**Figure 5:**
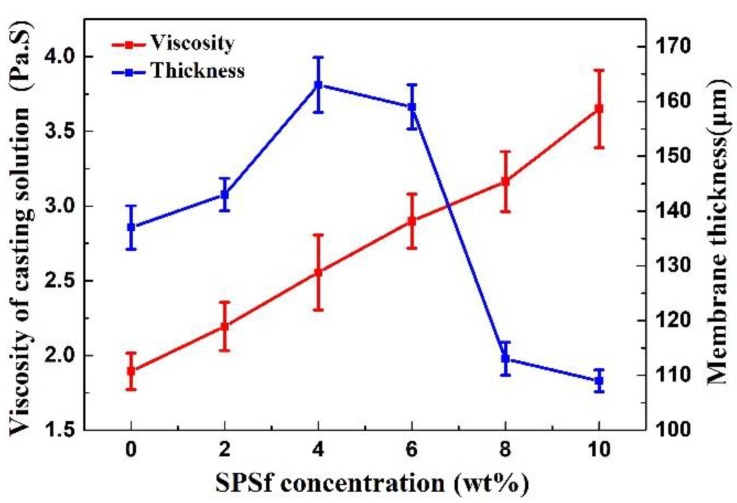
Effects of the SPSf concentration on the viscosity of the casting solution (temperature = 25 °C, rotation speed = 12 rpm) and the thickness of porous supports.

**Figure 6:**
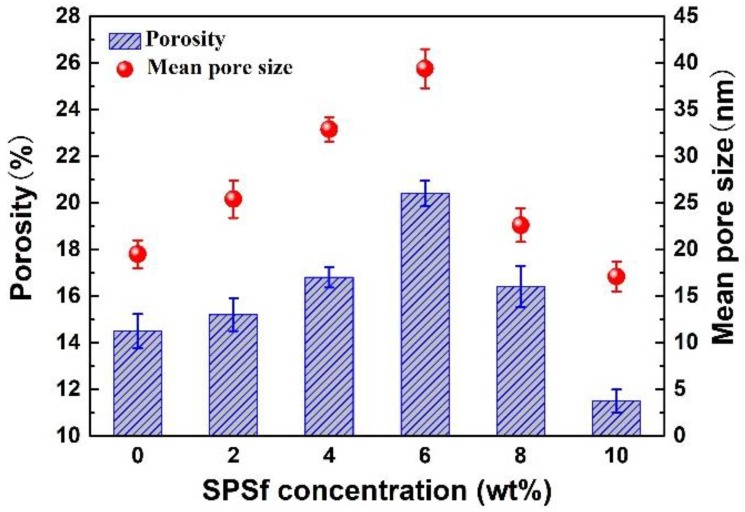
Surface porosity and mean pore size of the porous supports.

Rotor viscosimeter was used to test the viscosity. SEM was employed to observe the thickness and surface structure of the porous support (Figure S1). The results showed that the viscosity of casting solution increased with the increasing SPSf concentration (Figure 5), while the porosity, pore size, and pore thickness of the blended porous supports first increased with the rise in SPSf concentration less than 6 wt % and then decreased with further increase in SPSf concentration (Figure 5 and Figure 6).

#### 3.2.2. Surface Morphology and Wettability of the Membranes

**Figure 7:**
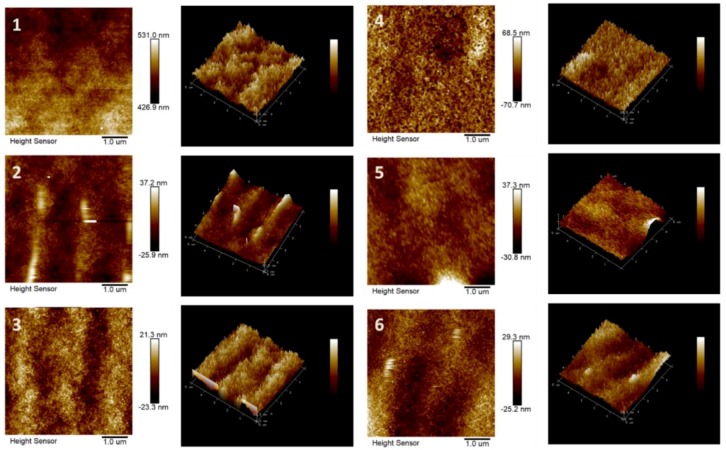
Surface AFM images of the 1–6 porous supports.

**Figure 8:**
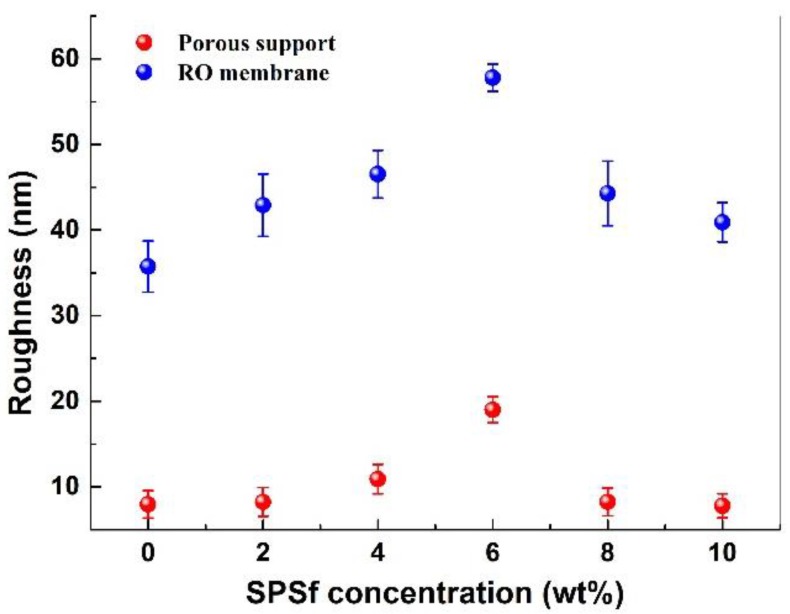
Surface roughness of the porous supports and the TFC RO membranes.

**Figure 9:**
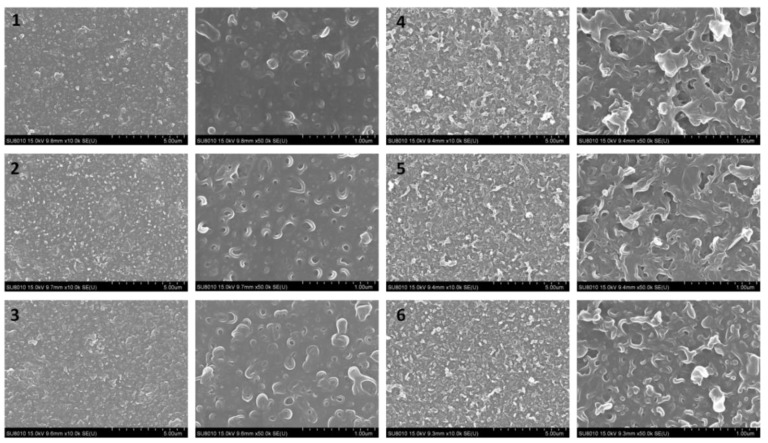
Surface SEM images of the polyamide TFC RO membranes prepared on 1–6 porous supports. The left images were magnified 10,000 times while the right images were magnified 50,000 times.

**Figure 10:**
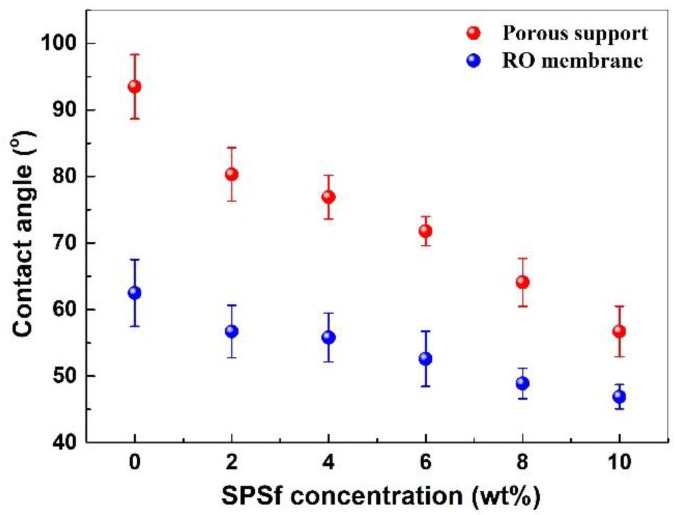
Contact angles of the porous supports and polyamide TFC RO membranes.

SEM and AFM were used to characterize the surface morphology of the porous support and RO membrane. The results showed that both the porous support and polyamide RO membrane surfaces exhibited increasing roughness with the increase in SPSf concentration up to 6 wt % but decreasing roughness at SPSf concentrations above 6 wt % (Figure 7, Figure 8 and Figure 9). However, their surface hydrophilicity always increased with increasing SPSf (Figure 10).

#### 3.2.3. Membrane Separation Performances

**Figure 11:**
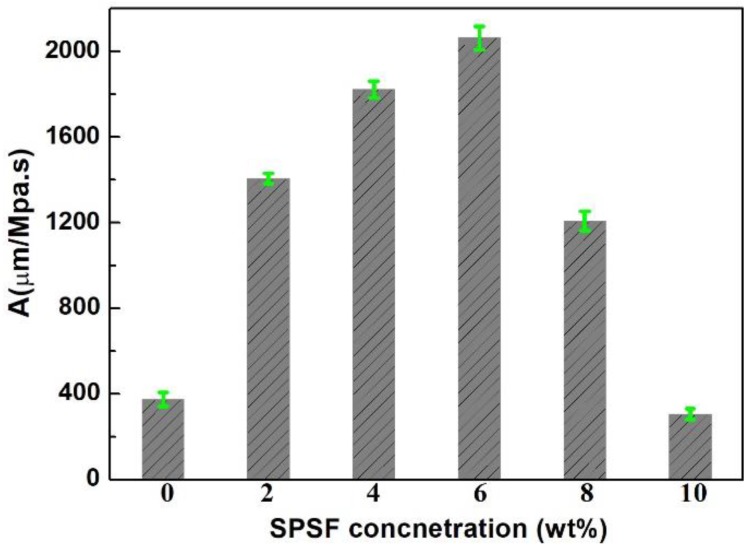
Permeability coefficients of the 1–6 porous supports.

**Figure 12:**
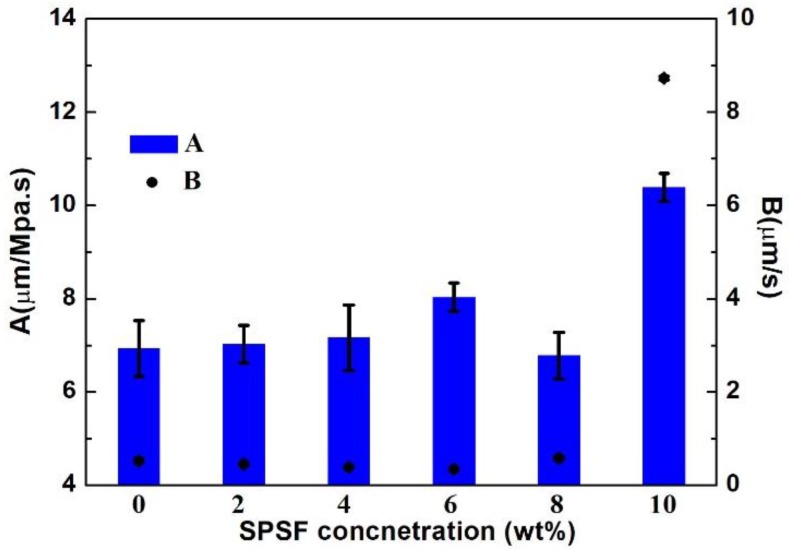
The separation performances of the polyamide TFC RO membranes.

The separation performances of the porous supports and polyamide RO membranes were evaluated. The results showed similar variation between the porous support and RO membrane. At 6 wt % SPSf, the porous support had the highest permeability coefficients (Figure 11) and correspondingly, the polyamide RO membrane based on this support had an optimal comprehensive separation performance (Figure 12).

In a word, the incorporation of SPSf adjusted the surface properties (the pore structure and the wettability) of the support through changing the phase inversion rate. Furthermore, the pore structure and surface hydrophilicity influenced the polyamide morphology together. Although the addition of SPSf made for higher porosity and bigger pore size, the hydrophilicity of the SPSf favored polyamide formation within the pore, which led to lower permeability. On the other hand, SPSf increased the swelling of the supports. Results show that the PSf/SPSf blended porous support fabricated with 6% SPSf and 14% PSf exhibited higher porosity, bigger pore diameter, and a rougher and more hydrophilic surface compared to the conventional PSf support, which was more beneficial to improving the polyamide TFC RO membrane’s separation performance.

## 4. Discussion

### 4.1. Effects of Solvent and Polymer Concentration of the Casting Solution on the Porous Support and Polyamide TFC RO Membrane

The solvent and polymer are crucial in the fabrication of the ultrafiltration support membrane due to their effects on the phase inversion process and the morphology of the UF membrane. Therefore, solvent type and polymer concentration were first investigated to confirm the optimal primary conditions for the fabrication of the support membrane. NMP, DMAc, and DMF were selected as the solvents for the casting solution, then different PSf porous supports and polyamide TFC RO membranes were fabricated by the phase inversion method and interfacial polymerization technology, respectively.

#### 4.1.1. Morphology of the PSf Porous Support and Polyamide RO Membrane

SEM was used to observe the surface and cross-sectional morphologies of the prepared PSf porous supports and corresponding TFC RO membranes, as shown in Figure 2. The cross-section images showed that all the porous supports exhibited inhomogeneous structures with a dense upside layer and different pore structures beneath the skin, which is consistent with previous studies [21,37]. The support prepared with NMP presented the largest cavities with irregular shape in breadth and length beneath the top skin, and the corresponding polyamide RO membrane exhibited a small nodular morphology and a smoother surface. The support fabricated with DMAc was full of regular extended finger-like cavities over the entire structure, and the corresponding polyamide RO membrane showed a typical ridge-and-valley structure and a relatively smooth surface. However, the support produced with DMF exhibited a fairly uniform spongy cross-section, and the corresponding RO membrane also presented a typical ridge-and-valley structure but with a rough surface.

#### 4.1.2. Separation Performance of the Porous Support and Polyamide TFC RO Membrane

The separation performances of the PSf supports and corresponding polyamide RO membranes fabricated with different solvents and PSf concentrations were tested, as shown in Figure 3 and Figure 4, respectively. It can be seen that the permeability coefficients of the PSf supports increased with different solvent in the order of NMP < DMAc < DMF, and decreased with the increasing PSf concentration in the casting solution. Obviously, DMF and low concentration of PSf were beneficial to the fabrication of a PSf porous support with high flux. The possible reason is that the viscosity of the casting solution increased with the increase of polymer concentration and the high viscosity caused the delayed exchange rate between solvent and non-solvent, which led to a thicker top layer, lower porosity, smaller pore radius, and less macrovoids in the support [38,39].

However, the impacts of different solvents and PSf concentrations on the polyamide TFC RO membranes differed from that on supports. According to Figure 4, the permeability coefficients of the RO membranes increased in order of DMF > NMP > DMAc, and first increased with the increasing PSf concentration in the range of 16–20 wt %, then decreased with the further increasing PSf concentration. The salt permeance is inverse. This can be explained by the casting solution penetrating through the polyester nonwoven fabric and more defects were formed on the PSf supports due to the low viscosity of the casting solution at low PSf concentrations, which damaged the separation performance of the resultant RO membranes. In other words, solvent DMF and 20 wt % PSf were beneficial to the fabrication of polyamide TFC RO membranes with ideal separation performance.

### 4.2. Effects of the Doping Content of SPSf in the Casting Solution on the Structure and Separation Performance of the Porous Support and Polyamide TFC RO Membrane

The structure and performance of the support was influenced by the SPSf concentration blended in the casting solution [40]. Consequently, changing the doping content of SPSf in the casting solution with DMF was proposed to regulate the nature and performance of the porous support and polyamide TFC RO membrane.

#### 4.2.1. Viscosity of the Casting Solution and the Thickness of the Porous Support

The viscosity of the casting solution was measured with a viscosity tester and the thickness of the porous support was measured with scanning electron microscopy. As presented in Figure 5, the casting solution’s viscosity increased with the increasing SPSf concentration, which led to an increase in membrane thickness at the low SPSf concentration (0–4 wt %). The possible reason was that the sulfonated acid groups of SPSf reacted with the solvent and impaired the solvent strength, thus improving the viscosity of the casting solution. With further increasing SPSf concentration, the increasing viscosity hindered the penetration rate of the non-solvent into the membrane matrix during the immersion step, in other words, the delayed phase separation caused the increase in membrane thickness. To further increase the SPSf concentration, the support thickness reduced rapidly due to the solubility of SPSf in water.

#### 4.2.2. Pore Nature of the Porous Support

Porosity and pore size are important parameters that characterize membrane structure and properties, which determine the permeability of ultrafiltration membrane. As shown in Figure 6, the surface porosity and pore size of PSf/SPSf supports increased initially, reaching maximum points of 20.4% and 39.3 nm, respectively, at 6 wt % SPSf. Further increasing the SPSf concentration decreased the porosity and the pore size. Obviously, the surface pore structure of the support was simultaneously affected by the hydrophilicity and the viscosity of the casting solution. At low SPSf concentrations (0–6 wt %), since the solubility of SPSf is higher than that of PSf, the accelerated inter-diffusion rate between SPSf and non-solvent during non-solvent-induced phase separation results in an increase of membrane porosity and pore size. With further increasing SPSf concentration from 6 wt % to 10 wt %, however, the surface porosity and pore size were reduced. The possible reason was that the viscosity of the casting solution increased sharply with the increasing SPSf concentration and the intermolecular entanglement was correspondingly intensified by macromolecular aggregation, meaning the solvent exchange was hindered. The higher the casting solution viscosity, the lower the occurrence of cavity formation during phase separation. As a result, the porosity and pore size decreased reversely at higher SPSf concentrations in the casting solution.

#### 4.2.3. Surface Morphologies of the Porous Supports and TFC RO Membranes

Atomic force microscopy (AFM) was used to characterize the surface roughness of different membranes. As shown in Figure 7 and Figure 8, with the increase of SPSf concentration from 0 to 6 wt %, the surface roughness (RMS) of the porous supports increased from 7.9 nm to 19.0 nm, while the roughness of the TFC RO membranes increased from 35.7 nm to 57.8 nm. With further increasing SPSf concentration from 6 wt % to 10 wt %, the roughness of the supports and polyamide RO membranes decreased ultimately to 7.8 nm and 40.9 nm, respectively. As mentioned in previous sections, at low SPSf concentrations (0–6%), the increasing demixing rate caused by the exchange between SPSf and non-solvent improved the surface porosity and pore size, which led to increasing roughness. At high SPSf concentration (8–10%), the sharply increasing viscosity hindered the rate of solvent and non-solvent exchange, resulting in less membrane shrinkage. Thus, the surface roughness decreased. Obviously, the result was consistent with the variation of surface pore structure as discussed above.

The conventional polyamide TFC membranes prepared by MPD-TMC interfacial polymerization exhibited typical ridge-and-valley structures, as shown in Figure 9. The polyamide TFC membranes prepared on the supports with lower concentration SPSf (0–4 wt %) exhibited more nodular morphology, but the surface of the other polyamide RO membranes fabricated on the supports with high concentration SPSf (6–10 wt %) presented leaf-like structure. It perhaps was due to (1) less MPD remaining in the support surface with lower porosity and smaller pore size. According to previous studies [41], the less MPD retain in the pore, the lower concentrations of MPD diffuse into hexane and then polymerize with TMC to form smaller nodular polyamides. With the increase of porosity and pore size, the more MPD remaining in the pores of the support layer can react with TMC to produce the leaf-like structure. (2) The more hydrophilic support is in favor of uniform MPD distribution and tends to form large leaf-like polyamides. It needs to be noted that MPD likely diffused more slowly out of the hydrophilic pores which gave rise to more polyamide formation deeper within support membrane pores. The cross-sectional images are displayed in Figure S2. It was nearly consistent with the nascent PSf support.

#### 4.2.4. Surface Wettability of the Porous Support and TFC RO Membrane

The contact angle is normally used to evaluate the wettability of the membrane surface. The lower the contact angle is, the more wettable the membrane surface is, and vice versa. Allowing for the correct determination of water contact angle on the porous support, the data was taken only as an indication of the membranes’ wettability trends, as in previous studies [21,23].

As can be seen from Figure 10, the surface contact angle of both the porous supports and corresponding polyamide TFC RO membranes reduced with the addition of SPSf, which was attributed to (1) the higher hydrophilicity of SPSf than PSf due to the sulfonic acid group; and (2) the increasing porosity and pore size caused by the solubility of SPSf as mentioned in Section 4.2.2. It is accepted that by increasing the pore size, the contact angle between the water and the porous surface reduces. In other words, both the supports and polyamide RO membranes exhibited surfaces with greater wettability due to the introduction of SPSf.

#### 4.2.5. Separation Performances of the Porous Support and TFC RO Membrane

The separation performances of the porous supports and corresponding RO membranes were tested and the results are shown in Figure 11 and Figure 12. It can be seen that the permeability coefficients of porous supports increased initially, reaching the maximum point of 2063 μm/MPa·s at 6 wt % SPSf and then decreased with further increasing SPSf concentration, which was in accord with the regularity of the pore structure of the porous supports.

According to Figure 12, the permeability coefficients of RO membranes fabricated with low SPSf concentrations (0–6 wt %) first increased slightly from 6.93 μm/MPa·s to 8.03 μm/MPa·s, while the permeability coefficients of RO membrane fabricated with 8 wt % of SPSf decreased to 6.78 μm/MPa·s, but the salt permeance of all these membranes were maintained at 0.3–0.5 μm/s. However, it was also noticed that the RO membrane fabricated with 10 wt % SPSf had the highest salt permeance of 8.73 μm/s. The possible reason was that the introduction of hydrophilic SPSf contributed to the improvement of surface hydrophilicity which was beneficial to the water flux of the membrane at relatively low SPSf concentrations (2–6 wt %). On the other hand, the SPSf-based membrane, especially with high SPSf concentration (10 wt %), swelled easily and caused defects on the polyamide layer, which caused a loss of salt rejection and increased water flux. In a word, the UF membrane with 6 wt % SPSf was the favorable porous support for fabrication of polyamide TFC RO membranes with better separation performance.

## 5. Conclusions

SPSf was used to modify conventional PSf ultrafiltration membranes to obtain suitable SPSf/PSf blended membranes which were further employed to fabricate aromatic polyamide TFC RO membranes with improved separation performance. The effects of solvent, polymer concentration, and doping content of SPSf in the casting solution on the structure and performance of both the porous support and RO membrane were systematically investigated. The results showed that DMF and 20 wt % polymer in the casting solution were more suitable for fabrication of the porous support. Meanwhile, at 6 wt % SPSf, the PSf/SPSf blended porous support had higher porosity, bigger pore diameter, a smoother and more hydrophilic surface, and higher water flux. On the basis of this resultant porous support, the corresponding polyamide RO membrane exhibited a more hydrophilic surface, leaf-like structure, and optimal separation performance. In a word, DMF, 6 wt % SPSf, and 14 wt % PSf were the optimal parameters for preparation of porous supports with ideal pore nature and performance that are beneficial to the fabrication of polyamide TFC RO membranes with favorable structure and separation performance.

## Figures and Tables

**Table 1:** Composition of the casting solution when fabricating the porous supports.

No.	PSf (wt %)	SPSf (wt %)	Solvent (wt %)
1	20	0	80
2	18	2	80
3	16	4	80
4	14	6	80
5	12	8	80
6	10	10	80

**Table 2:** Composition of the aqueous phase and organic phase when preparing the TFC RO membrane.

Solution	Composition
Aqueous phase	2 wt % MPD, 2 wt % TEA, 0.15 wt % SDS and 4 wt % CSA in water
Organic phase	0.15 wt % TMC in hexane

## Supplementary Materials

The following are available online at http://www.mdpi.com/2073-4360/10/6/686/s1, Figure S1: Surface SEM images of the 1–6 substrates and selected area for image analysis; Figure S2: Cross-section images of the 1–6 porous supports.
