# Supplementary material for: Modification of PSf/SPSf Blended Porous Support for Improving the Reverse Osmosis Performance of Aromatic Polyamide Thin Film Composite Membranes
Source: Polymers (Basel). 2018 Jun 20;10(6):686. doi: 10.3390/polym10060686 (PMC6404368; doi:10.3390/polym10060686)
Supplement: Supplementary file 1 [file polymers-10-00686-s001.pdf]

Supplementary Materials

Modification of PSf/SPSf Blended Porous Support for Improving the Reverse Osmosis Performance of Aromatic Polyamide Thin Film Composite Membranes

Li-Fen Liu [1,2,*], Xing-Ling Gu [1], Xin Xie [1], Rui-Han Li [1], Chun-Yang Yu [3], Xiao-Xiao Song [1,2,*] and Cong-Jie Gao [1,2]

[1] Center for Membrane and Water Science and Technology, Ocean College, Zhejiang University of Technology, Hangzhou 310014, China; 13588348159@163.com (X.-L.G.); 15958041452@163.com (X.X.); 15857112095@163.com (R.-H.L.); gaocj@zjut.edu.cn (C.-J.G.)

[2] Collaborative Innovation Center of Membrane Separation and Water Treatment of Zhejiang Province, Hangzhou 310014, China

[3] State Key Laboratory of Metal Matrix Composites, School of Chemistry & Chemical Engineering, Shanghai Jiao Tong University, 800 Dongchuan Road, Shanghai 200240, China; chunyangyu@sjtu.edu.cn

* Correspondence: lifenliu@zjut.edu.cn (L.-F.L.); songxiaoxiao@zjut.edu.cn (X.-X.S.); Tel.: +86-571-88325373 (L.-F.L.)

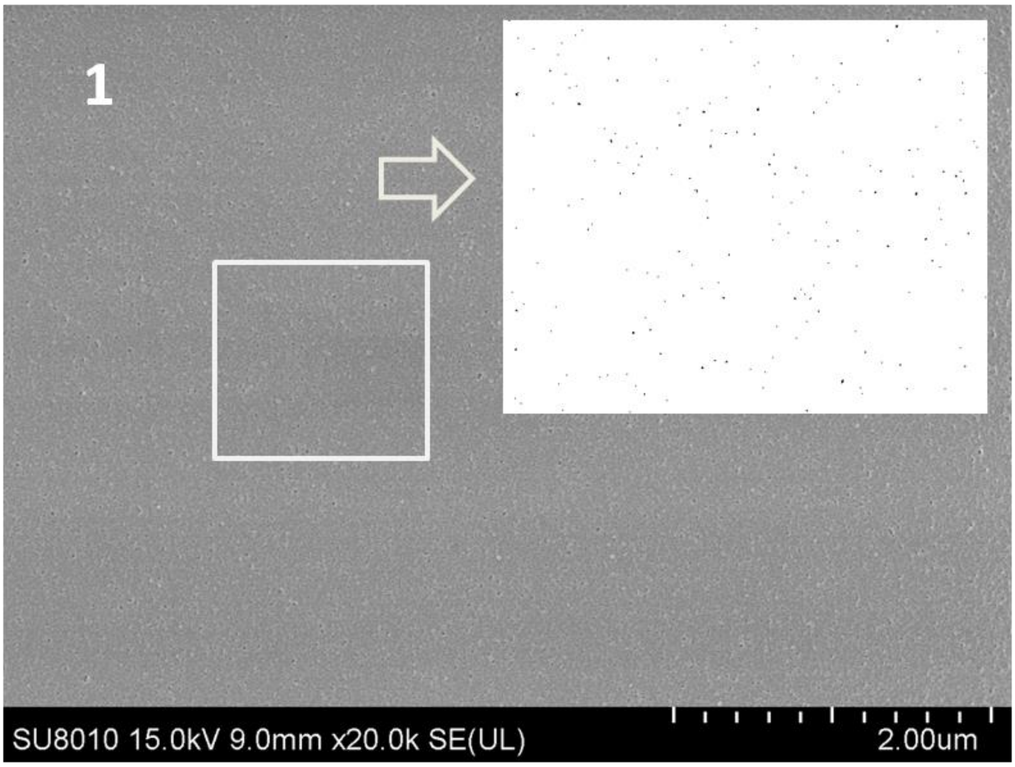

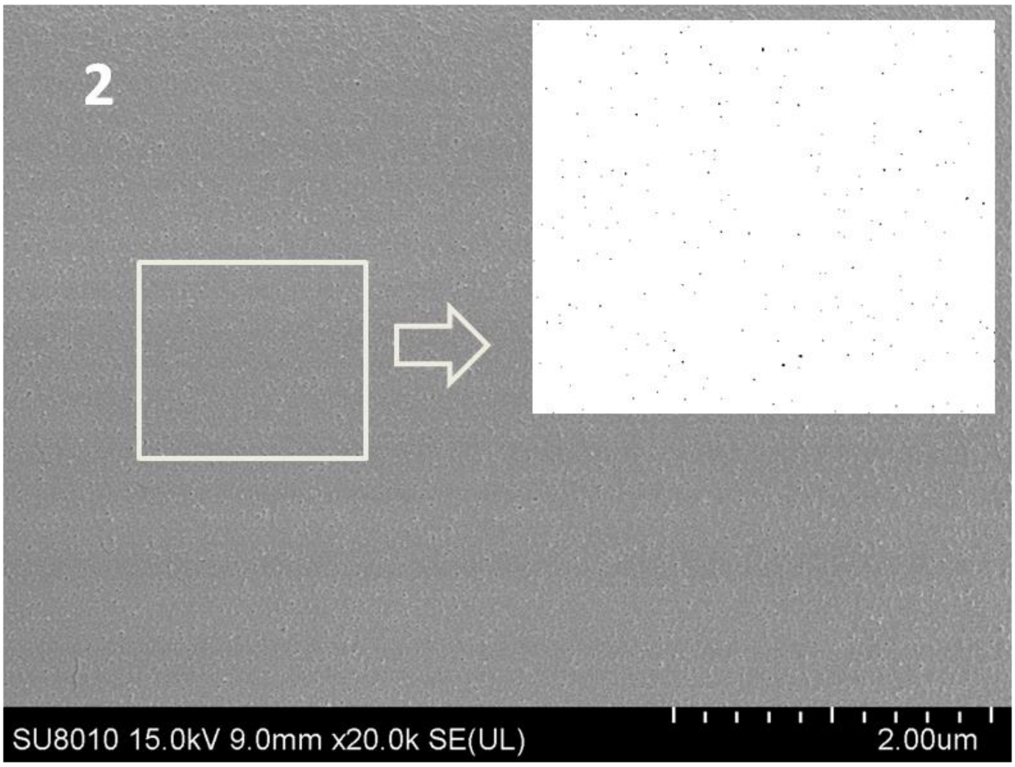
2
SU8010 15.0kV 9.0mm x20.0k SE(UL)
2.00um

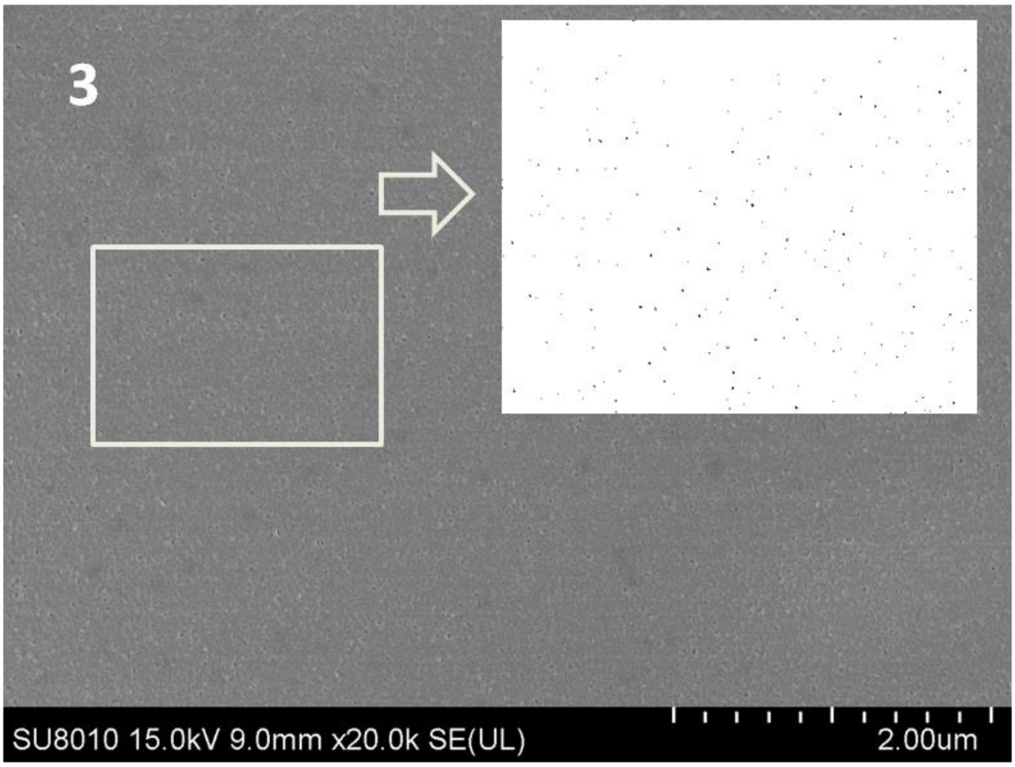
3
SU8010 15.0kV 9.0mm x20.0k SE(UL)
2.00um

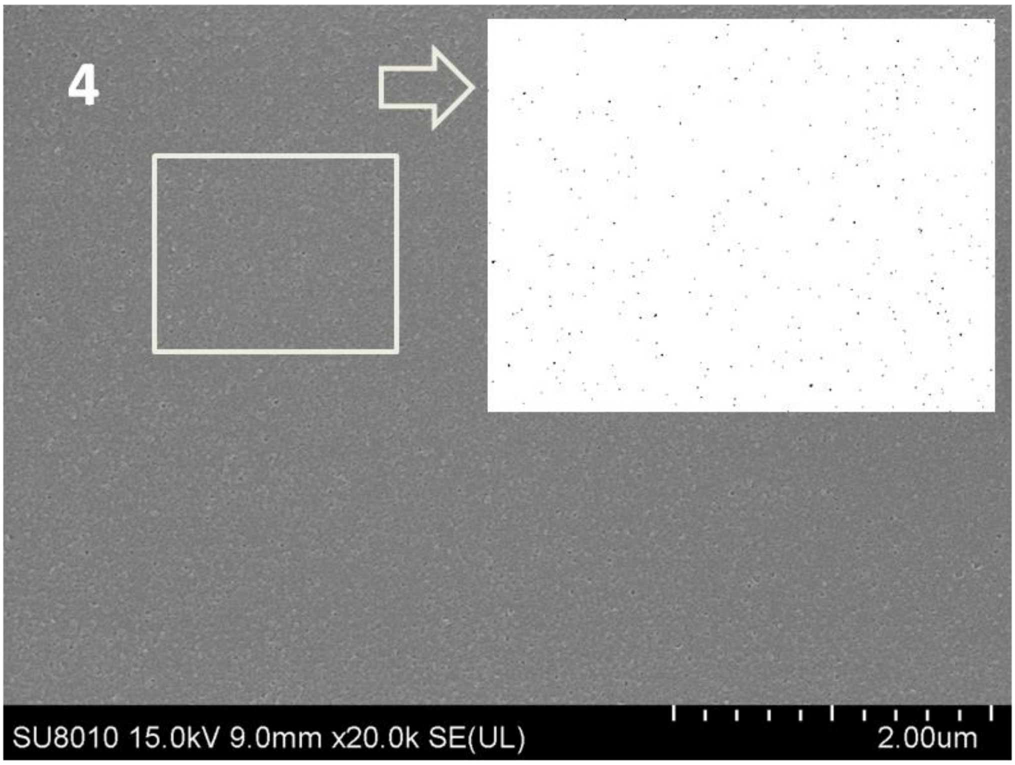
4
SU8010 15.0kV 9.0mm x20.0k SE(UL)
2.00um

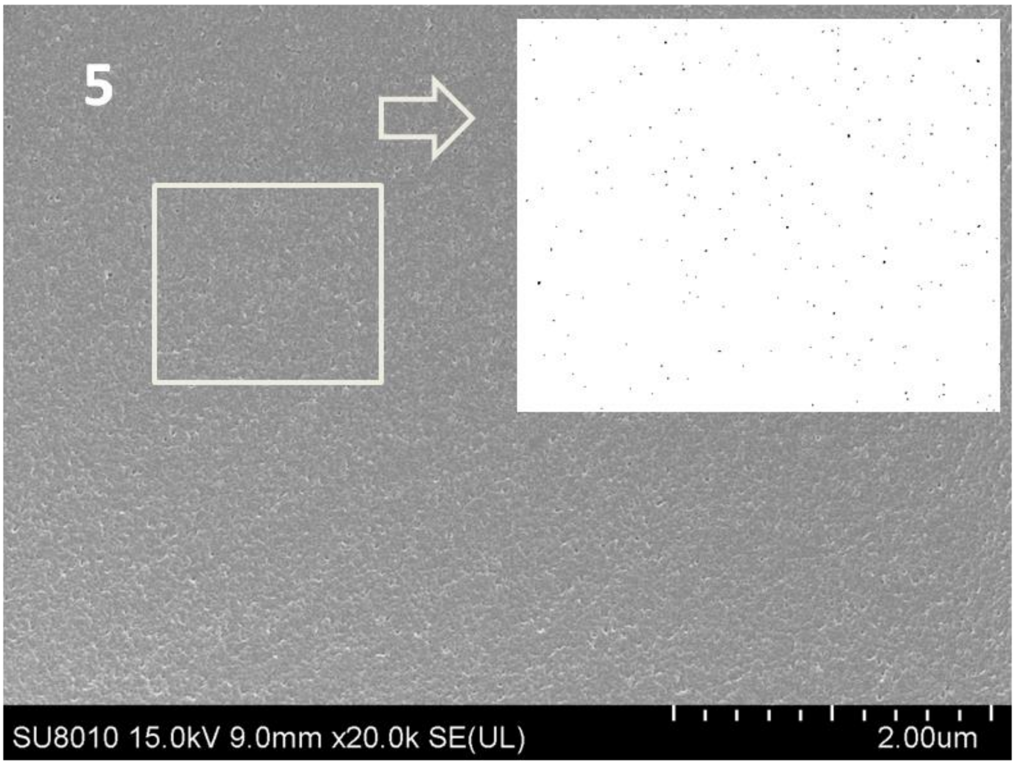
5
SU8010 15.0kV 9.0mm x20.0k SE(UL)
2.00um

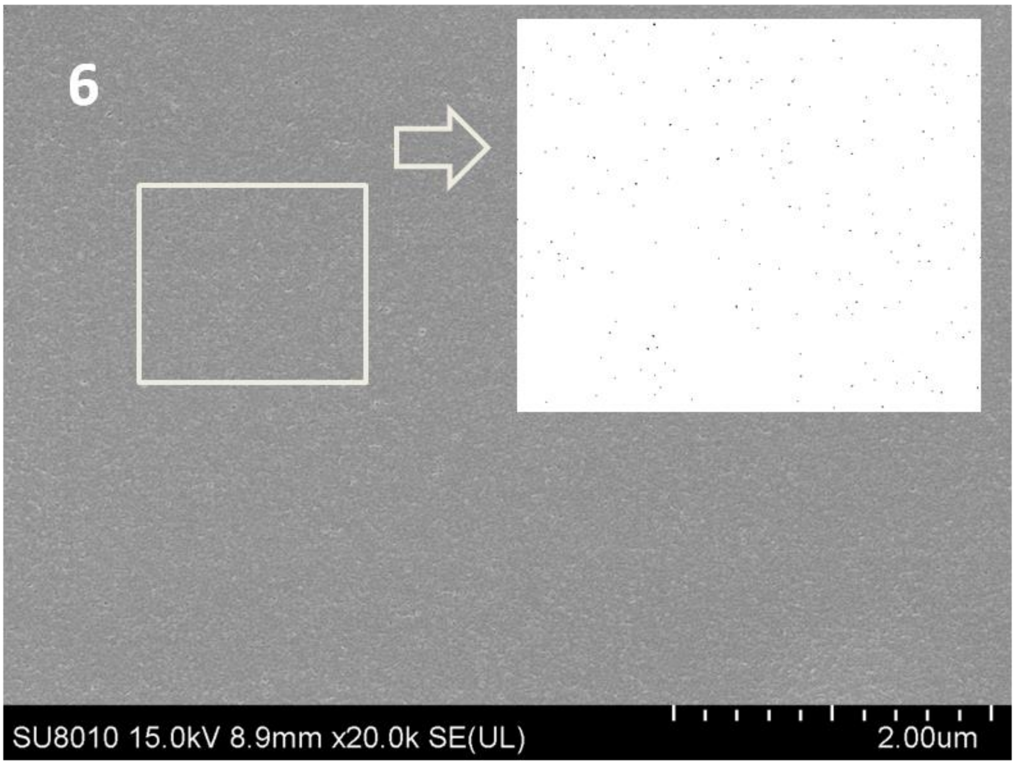

Figure S1. Surface SEM images of the 1–6 substrates and selected area for image analysis.

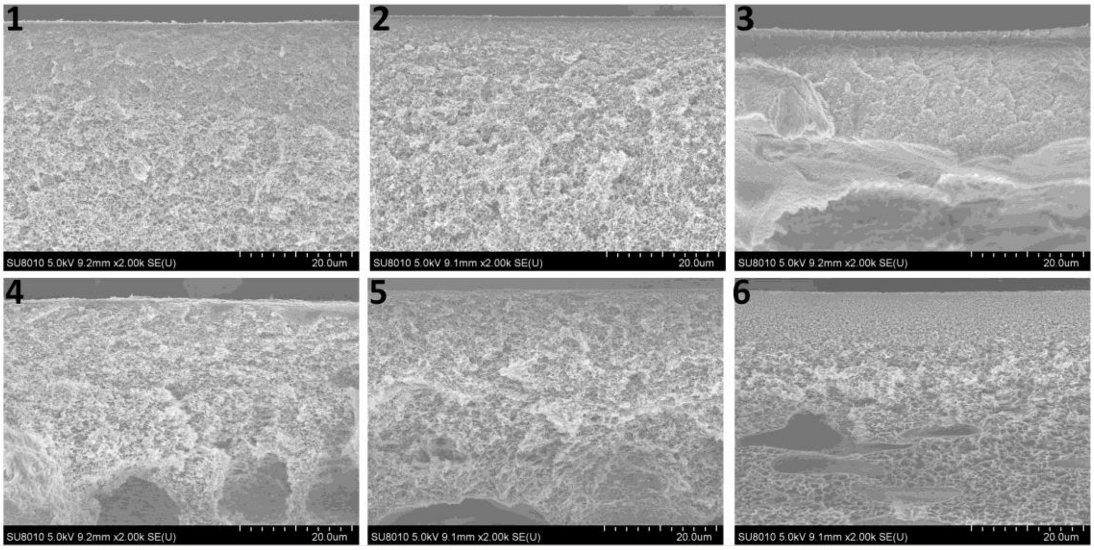

Figure S2. Cross-section images of the 1–6 porous supports.
